# Comparison of the detection of periodontal pathogens in bacteraemia after tooth brushing by culture and molecular techniques

**DOI:** 10.4317/medoral.20842

**Published:** 2016-03-06

**Authors:** María-Jose Marín, Elena Figuero, Itziar González, Ana O´Connor, Pedro Diz, Maximiliano Álvarez, David Herrera, Mariano Sanz

**Affiliations:** 1 PhD in Pharmacy. Researcher. Oral Research Laboratory, Faculty of Odontology, University Complutense, Madrid, Spain; 2 DDS, PhD. Assistant Doctor Professor of Periodontology, Department of Stomatology III, Faculty of Odontology, University Complutense, Madrid, Spain; 3 PhD in Pharmacy. Technician. Oral Research Laboratory, Faculty of Odontology, University Complutense, Madrid, Spain; 4 Technician. Oral Research Laboratory, Faculty of Odontology, University Complutense, Madrid, Spain; 5 MD, DDS, PhD. Profesor of Periodontology, School of Medicine and Dentistry, Santiago de Compostela University, Spain; 6 PhD in Medicine: Microbiology Specialist in Research Laboratory, Department of Clinical Microbiology, Xeral-Cíes Hospital, Vigo, Spain; 7 DDS, PhD. Associate of Periodontics, Department of Stomatology III, Faculty of Odontology, University Complutense, Madrid, Spain; 8 MD, DDS, PhD. Professor of Periodontology, Department of Stomatology III, Faculty of Odontology, University Complutense, Madrid, Spain

## Abstract

**Background:**

The prevalence and amounts of periodontal pathogens detected in bacteraemia samples after tooth brushing-induced by means of four diagnostic technique, three based on culture and one in a molecular-based technique, have been compared in this study.

**Material and Methods:**

Blood samples were collected from thirty-six subjects with different periodontal status (17 were healthy, 10 with gingivitis and 9 with periodontitis) at baseline and 2 minutes after tooth brushing. Each sample was analyzed by three culture-based methods [direct anaerobic culturing (DAC), hemo-culture (BACTEC), and lysis-centrifugation (LC)] and one molecular-based technique [quantitative polymerase chain reaction (qPCR)]. With culture any bacterial isolate was detected and quantified, while with qPCR only *Porphyromonas gingivalis* and *Aggregatibacter actinomycetemcomitans* were detected and quantified. Descriptive analyses, ANOVA and Chi-squared tests, were performed.

**Results:**

Neither BACTEC nor qPCR detected any type of bacteria in the blood samples. Only LC (2.7%) and DAC (8.3%) detected bacteraemia, although not in the same patients. *Fusobacterium nucleatum* was the most frequently detected bacterial species.

**Conclusions:**

The disparity in the results when the same samples were analyzed with four different microbiological detection methods highlights the need for a proper validation of the methodology to detect periodontal pathogens in bacteraemia samples, mainly when the presence of periodontal pathogens in blood samples after tooth brushing was very seldom.

**Key words:**Bacteraemia, periodontitis, culture, PCR, tooth brushing.

## Introduction

Periodontitis is a chronic inflammatory disease triggered by bacterial species residing in the subgingival biofilm. Among these bacteria, *Porphyromonas gingivalis, Aggregatibacter actinomycetemcomitans*and *Tannerella forsythia*have shown the highest level of association (1) although the use of current molecular based diagnostic methods has shown that there is a high proportion of uncultivable bacteria within the biofilm, whose relevance we do not know (2). In gingivitis and periodontitis, the sub gingival biofilm is in close proximity with the highly inflamed gingival marginal tissues, where the epithelium is ulcerated and the underlying connective tissue is highly vascularized, what results in an easy portal of entry for bacterial species into the blood circulation (bacteraemia) and possible spread to distant organs (3). This mechanism has been attributed as one of the key processes explaining the associations between periodontitis and systemic diseases such as cardiovascular diseases (4).

Several studies have reported that bacteraemia occurs more frequently immediately after various preventive and therapeutic oral procedures such as scaling and root planing, periodontal probing or periodontal surgery (5), although routine daily activities, such as chewing or tooth brushing may also cause low-grade bacteraemias with likely systemic dissemination of these bacteria (6) leading to a chronic cumulative effect, which may be even more pathogenic than isolated events in relation to clinical procedures (5,7). There is, however, limited information on the systemic consequences of low-grade bacteraemia, even though they have been demonstrated after chewing (8,9), tooth brushing, (8,10-14) or after dental flossing (15). The results from these studies are very heterogeneous in terms of prevalence (0-90%), with some reporting the presence of periodontal pathogens, such as *A. actinomycetemcomitans*(15,16), while others were not able to detect them using similar diagnostic techniques (8,9,11,13,17,18).

In these studies the most frequently used detection method was hemo-culture by BACTEC® (Becton, Dickinson and Company, Franklin Lakes, NJ, USA), a method that is still in current use in many clinical microbiology laboratories (19). This automated culture method is designed to qualitatively detect microbial growth in blood specimens. It uses a continuous-monitoring instrument that agitates and incubates BACTEC® blood culture bottles, detecting increases in CO2 produced by microbial growth through a noninvasive fluorescent technology. The process includes a subsequent sub culturing on agar media to identify the isolates. Another frequently used culture-based method is the lysis-filtration (LF) where the patient´s blood is first lysed, then filtered through a membrane and then transferred to a culture broth. The lysis-centrifugation (LC) method is a similar technique where the blood is first centrifuged, filtered and then conventional agar media is used for the isolation, identification and quantification of the detected bacteria (20). Even though these modified culturing techniques have been specifically designed for blood samples, direct anaerobic culturing (DAC) may also be used for detecting and counting the number of colonies present by inoculating the blood sample directly onto adequate plate media and appropriate culture conditions (21).

Finally, quantitative polymerase chain reaction (qPCR) is a molecular method that enables the detection of very small amounts of the targeted DNA (not depending on bacterial growth), and hence, it may also allow for detecting low levels of targeted bacteria. In fact, previous studies using qPCR technology have shown a high sensitivity and specificity for detecting and quantifying target microorganisms, not only in subgingival plaque samples, but also in vascular and blood samples (22).

Beyond the possible variability derived from the different microbiological techniques used for their detection and quantification, the reported differences in bacteraemia studies may also be due to differences in the subject periodontal health status or to differences in the triggering event (daily life activity or oral intervention). All these potential sources of variability make the interpretation of bacteraemia studies difficult and hence, it is difficult to attribute the relative importance of this mechanism in the pathogenesis of the demonstrated associations between periodontitis and systemic diseases.

It is, therefore, the purpose of this methodological comparative study to validate the hypothesis that similar bacteraemia results would be obtained irrespective from the diagnostic technique used (BACTEC, LC, DAC and qPCR) in serum samples obtained from subjects with different periodontal conditions immediately harvested after tooth brushing.

## Material and Methods

- Study design

This cross-sectional study was approved by the Regional Committee on Ethics in Research of Galicia (Spain). All subjects participating agreed by signing a written informed consent and the study procedures were conducted according to the Declaration of Helsinki, the Council of Europe Convention, the Universal Declaration of UNESCO and the requirements of the Spanish legislation.

- Patient selection

A convenience sample of subjects volunteered for this investigation were consecutively selected between January and April 2012 among postgraduate students and those attending the dental clinics at the Faculty of Odontology in the University of Santiago de Compostela (Spain). Exclusion criteria included the following: (a) fewer than 20 natural teeth; (b) use of systemic antibiotics within the previous 3 months; (c) use of oral antiseptics routinely within the previous 3 months; (d) congenital or acquired immune deficiency or any other disease that would facilitate the development of infections; (e) bleeding complications; (f) other relevant systemic disease (hematological disorders, congenital or acquired heart defects, diabetes and pregnancy) and (g) previous problems with venipuncture.

- Study visits

In the first visit, each patient received a periodontal and radiographic examination, including gingival index (GI), probing pocket depth (PPD), clinical attachment loss (CAL), bleeding on probing (BoP) and periapical x-rays. All clinical outcome variables were recorded by one experienced operator (PD).

From this examination, the periodontal status of the included subjects was defined according to the following criteria (23):

- Healthy: less than three sites with PPD≥ 3 mm or CAL ≥2 mm, no radiographic evidence of bone loss and GI≤1.

- Gingivitis: less than three sites with PPD≥ 3 mm or CAL ≥2 mm, and no radiographic evidence of bone loss, with GI≥ 1.

- Periodontitis: at least one site per quadrant with PPD≥5 mm, BoP and CAL≥3 mm, together with generalized radiographic bone loss greater than 30%.

After the clinical and radiological procedures, subgingival samples were taken.

During the second visit, participants performed a supervised tooth brushing session during 2 minutes using a medium hardness toothbrush (Vitis Access® Medio, Dentaid, Barcelona, Spain) and applying the Bass technique. Peripheral blood samples were drawn by one experienced nursing assistant before and immediately after tooth brushing by venipuncture under standardized conditions.

- Subgingival plaque samples

In healthy subjects, subgingival samples were taken from the mesio-buccal sites of the first molars and, when absent, from the adjacent second molars (the next alternative would be the second premolars and from there, any teeth present mesially). In subjects with gingivitis or periodontitis, subgingival samples were taken from the most accessible site with the deepest PPD and/or BoP, per quadrant. Samples were taken with two consecutive sterile medium paper points (Maillefer, Ballaigues, Switzerland) (24). Prior to sampling, the selected sites were isolated from saliva and supragingival contamination with the use of cotton rolls and compressed air. The paper points were inserted subgingivally, kept in place for 10 s and then transferred into a screw-capped vial, containing 1.5 mL of reduced transport fluid (RTF). These vials were sent to the Laboratory of Research, University Complutense, Madrid (Spain) within 24 h, where they were processed by two experienced lab technicians (IG, AO). In the laboratory the samples were homogenized by vortexing for 30 s (25), and serially diluted in phosphate buffer saline (PBS). Aliquots of 0.1 mL were then plated manually using specific medium Dentaid-1 for detection of *A. actinomycetemcomitans*(26) and incubated for 3 days in air with 5% CO2 at 37ºC. Suspected isolates were identified on the basis of colony morphology (small colony, 1 mm in diameter, with a dark border and a “star” or “crossed cigars” shaped inner structure) and positive catalase reaction. Sample dilutions were also plated onto a non-selective blood agar plate (Blood Agar Base II®, Oxoid, Basingstoke, England), supplemented with haemine (5 mg/L), menadione (1 mg/L) and 5% sterile horse blood. After 7-14 days of anaerobic incubation (80%N2, 10% CO2 and 10% H2), total counts and counts of representative colonies (those with colony morphologies compatible with target pathogen morphology) were carried out in selected plates (those having between 30-300 colonies). Suspected colonies were further identified by microscopy, gram-staining and enzyme activity (including N-acetyl-β-D-glucosaminidase, α-glucosidase, α-galactosidase, α-fucosidase, esculin, indole and trypsin-like activity). Counts were transformed in colony forming units (CFU) per mL and total anaerobic counts were calculated, as well as counts of the detected periodontal pathogens (*A. actinomycetemcomitans, T. forsythia, P. gingivalis, Prevotella intermedia/nigrescens, Parvimonas micra, Campylobacter rectus* and *Fusobacterium nucleatum*). In addition to the quantitative microbiological data, the frequency of detection and proportions for each bacterial species were also calculated.

- Blood samples

After disinfection with alcohol and povidone iodine, an intravenous catheter was inserted into the antecubital fossa or on the dorsum of the hand using an angiocath® (18-22 G, Becton Dickinson, Spa USA). Peripheral venous blood samples were collected from each patient before tooth brushing and 30 s after tooth brushing.

To minimize the risk of bacterial contamination, the venous cannula was flushed with 3 mL of saline after each blood collection and the first 2 mL of blood drawn was discarded (8,16). The collection was performed in Isolator tubes® (Oxoid Limited, Basingstoke, Hants, United Kingdom) for the lysis-centrifugation technique and in tubes with ethylene-diamine-tetra-acetic acid (EDTA) (Vacutainer®, Becton Dickinson, San Agustín de Guadalix, Madrid, Spain) for the other techniques, following the recommendations of the Spanish Society of Clinical Microbiology and Infectious Diseases (8).

The blood samples from each subject were analyzed using four different techniques.

- BACTEC. 5 mL of blood from EDTA tubes were inoculated into containers with aerobic and anaerobic culture media (BACTEC plus®) and immediately transported to the Department of Microbiology of Complejo Hospitalario Universitario de Vigo-CHUVI (Vigo, Spain) for analysis within 24 hours, by an experienced microbiology specialist (MA),

- LC. Isolator tubes containing 10 mL of blood were processed in the Microbiology Laboratory, Faculty of Medicine and Odontology, University of Santiago de Compostela (Spain), by an experienced researched (PD).

- DAC and qPCR. EDTA tubes were immediately sent to the Laboratory of Research, Faculty of Odontology, University Complutense, Madrid (Spain), and 1 mL of blood was used for each technique. The procedures were carried out by an experienced researched (MJM).

BACTEC blood culture. BACTEC® bottles were incubated and continuously monitored over 14 days for the presence of microorganisms in BACTEC 9240® (Becton, Dickinson and Company, Franklin Lakes, NJ, USA). A Gram stain was performed on each positive blood culture. Positive aerobic blood cultures were subcultured on blood agar in anaerobiosis, on chocolate agar medium in an atmosphere with 5-10% CO2 and on MacConkey agar in an aerobic atmosphere. The same protocol was used for positive anaerobic blood cultures, although also including subculture on Schaedler agar incubated in an anaerobic atmosphere (80%N2, 10% CO2 and 10% H2). Isolated bacteria were identified using a battery of biochemical tests provided by the Vitek system (BioMérieux Inc., Hazelwood, MO, USA) for Gram-positive bacteria, *Neisseria spp./Haemophilus spp.*and strict anaerobic bacteria.

Lysis-centrifugation culture (LC). LC tubes (Oxoid Limited, Basingstoke, Hants, United Kingdom) were inoculated with each blood sample immediately after collection. Then tubes were firstly shaken, placed in a rack for 20 min and centrifuged at 1500 rpm for 30 min. Following removal of the supernatant, the pellet containing the material to be cultured was re-suspended in the remaining liquid and equal amounts from each tube were then plated onto chocolate and Schaedler agar media. The chocolate agar plates were incubated for 7 days with CO2 and Schaedler agar plates were incubated for 10 days in anaerobic atmosphere. If growth on the plates was observed, bacteria were identified using conventional biochemical tests.

Direct anaerobic culture (DAC). Similar to subgingival samples, blood samples were cultivated on agar-blood medium incubated for 7-14 days in jars in an anaerobic atmosphere and on selective medium Dentaid-1 incubated for 3-5 days in 5% carbon dioxide. The identification method was similar to the procedures reported for subgingival samples.

Quantitative PCR (qPCR) for *P. gingivalis*and *A. actinomycetemcomitans*. Bacterial DNA was extracted using a commercial kit specifically designed to extract bacterial DNA in blood samples (MoIYsis Complete5. Molzym Gmbh & Co.KG. Bremen, Germany) following manufacturer’s instructions.

The sequence of the primers and probes used for *P. gingivalis* and *A. actinomycetemcomitans* has been previously published by Boutaga *et al.* (27,28). In all cases, primers and probe sequences targeted 16S ribosomal-RNA (rRNA) gens ( Table 1). The oligonucleotide probes were labelled with the fluorescent dyes 6-carboxyfluorescein (FAM) at the 5-end in all cases and 6-carboxytetramethylrhodamine (TAMRA) at the 3- end for *P. gingivalis* and *A. actinomycetemcomitans.*

**Table 1:**
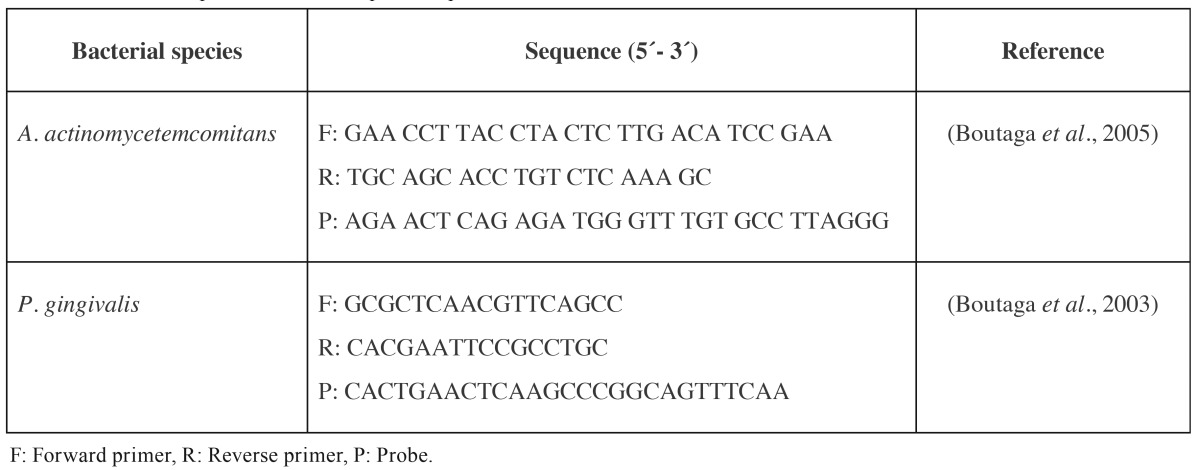
Primers and probe used for the qPCR amplifications.

The hydrolysis probes 5´nuclease assay PCR method was used for detecting and quantifying the bacterial DNA. PCR amplification was performed in a total reaction mixture volume of 20 µL. The reaction mixtures contained 10 µL of 2x TaqMan master mixture, optimal concentrations of primers and probe (300, 300 and 100 nM for *A. actinomycetemcomitans;* 300, 300 and 300 nM, for *P. gingivalis*), and 5 µL of DNA from the samples. The no-template control (NTC) consisted on 5 µL of sterile water. Samples were subjected to an initial amplification cycle of 95°C for 10 min, followed by 45 cycles at 95°C for 15 s and 60°C for 1 min in a thermocycler. Quantification cycle (Cq) values, previously known as cycle threshold (Ct) values, describing the PCR cycle number at which fluorescence rises above the baseline, were determined. Quantification was based on standard curves, which were constructed by plotting Cq values generated from qPCR against *P. gingivalis* and *A. actinomycetemcomitans* (log CFU mL-1). To prevent potential false-positive results, the limit of detection was calculated using the Cq value from the last point of the standard curve that holds 5 units of difference with the lowest Cq value from the NTCs obtained throughout experiments.

All assays were performed using calibration curves with a linear quantitative detection range established by a slope range 3.3-3.7 cycles/log decade, r2>0.997 and an efficiency range of 1.9-2.0.

Statistical analyses

Sample size calculation was based in previous data regarding tooth brushing-induced bacteraemia (8,10-13,17).

The primary outcome variable was the presence/absence of bacteraemia. Secondary outcome variables include all other microbiological variables including: total anaerobic counts, frequency of detection of target pathogens, counts of each studied pathogen and proportions of flora of each pathogen.

Total anaerobic counts, proportions of flora and pathogen counts were compared with ANOVA as described for the clinical variables. Frequencies of detection were compared using the chi-square tests. The level of statistical significance was set at *p*<0.05.

## Results

- Sample description

The screening included 50 subjects, but six did not satisfy the selection criteria, five were reluctant to participate and, in three subjects blood sampling was not complete due to the complain of the patient (anxiety or faint). Therefore, a sample population of 36 subjects was included, with 18 males and 27 females, and with a mean age of 29.7 ± 4.7 years (range, 23-55 years). According to the periodontal status, 17 were classified as periodontally healthy, 10 as gingivitis and 9 with moderate-severe periodontitis.

- Subgingival microflora

One of the subgingival samples could not be analyzed due to technical reasons.

 Table 2 depicts the data on detection of pathogens from subgingival samples in the three clinical categories, including their frequency of detection, mean proportions and mean amount of CFU/mL. *A. actinomycetemcomitans* was not detected in any of the subgingival samples. *P. intermedia* and *F. nucleatum* were the most prevalent bacterial species found in periodontal subjects (88%), followed by *P. gingivalis* (75%). *F. nucleatum* was the most common periodontal bacteria identified in gingivitis (90%) and in healthy (59%) patients. Differences among the study groups were statistically significant for *P. intermedia* (*p*=0.004) and *P. gingivalis* (*p*≤0.001).

**Table 2:**
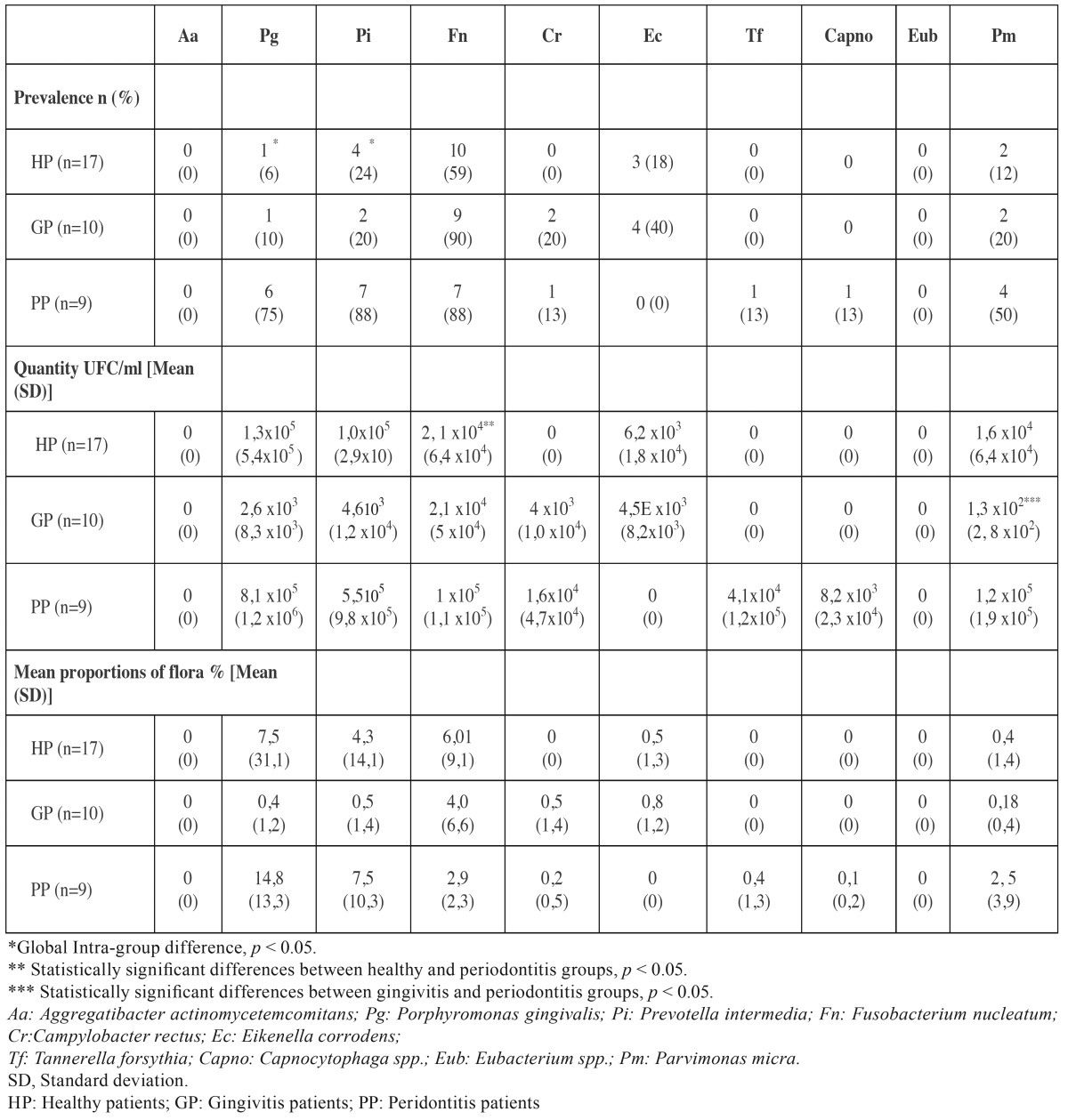
Frequency of detection (%), mean counts (in colony forming units, CFU/mL) and mean proportions of flora (%) of periodontal pathogens in subgingival samples according to periodontal status.

The mean amount of the total microflora in subgingival samples was 4.6x105 (standard deviation, SD=1.2x106) CFU/mL in healthy individuals, 7.18x105 (SD=1.2x106) CFU/mL in gingivitis patients and 4.4x106 (SD=3.9x106) CFU/mL in periodontitis patients, being these differences statistically significant between periodontitis and both healthy and gingivitis patients (*p*≤0.001; *p*=0.002, respectively). The highest counts were found for *P. gingivalis, P. intermedia* and *P. micra* (1x104-1x105 CFU/mL). Statistically significant differences were observed for *P. micra*between gingivitis and periodontitis patients (*p*=0.045) and for *F. nucleatum* between healthy and periodontitis patients (*p*=0.050).

*P. gingivalis* and *P. intermedia* proportions were higher in periodontitis patients than in healthy and gingivitis patients, while *F. nucleatum* proportions were higher in healthy patients, although these differences in proportions were not statistically significant.

- Bacteraemia

 Table 3, Table 4 depict the data obtained from the analysis of the blood samples with the different microbiological techniques.

**Table 3:**
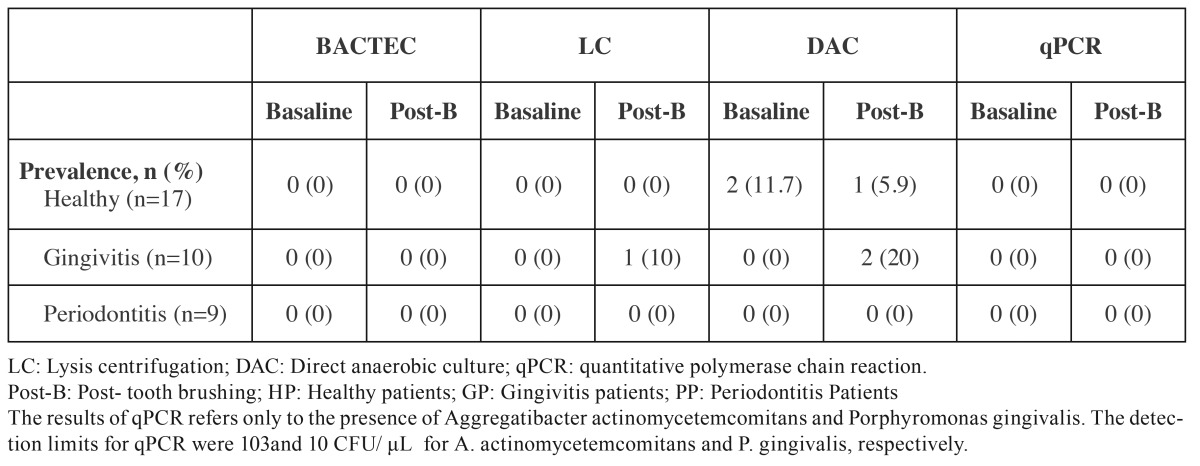
Frequency of detection of bacteraemia in positive samples by different techniques, according to periodontal status.

**Table 4:**
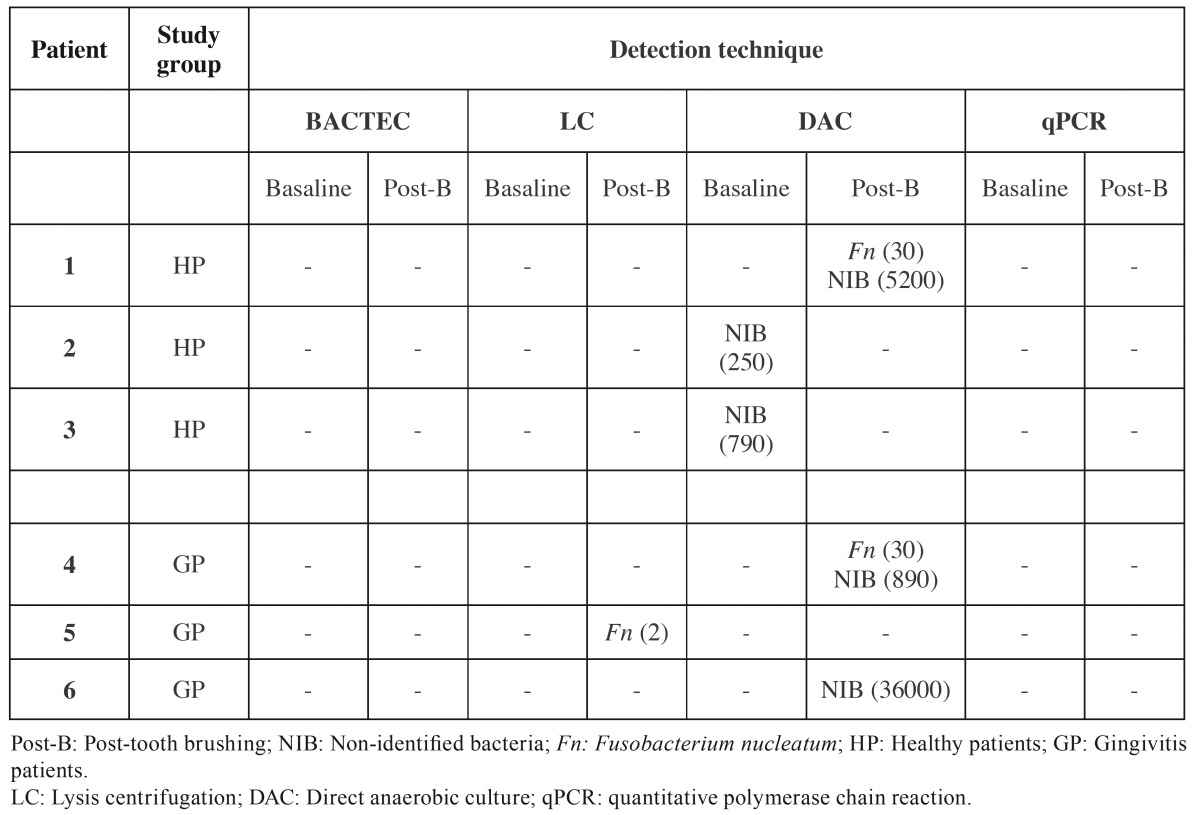
Detection and amount of CFU/mL (in parenthesis) of pathogens in positive blood samples obtained by different technics, according to periodontal status.

Neither BACTEC nor qPCR (only targeted to *A. actinomycetemcomitans * and *P. gingivalis*) detected any type of bacteria in any of the blood samples. LC and DAC detected bacteraemia, although not in the same samples. With LC, *F. nucleatum* was identified in low concentration (2 CFU/mL) in one sample after brushing from a patient with gingivitis (10%). With DAC, two healthy patients (11.7%) had non-identified bacteria (NIB) in basal condition and after tooth brushing, while one healthy patient (5.9%) and two gingivitis subjects (20%) had *F. nucleatum* (30 CFU/mL) and NIB.

*Staphylococcus spp., Bacillus spp., Corynebacterium spp., Micrococcus spp.* and *Moraxella spp.* were also identified by BACTEC system or LC, although they were considered contaminants from either the blood sampling procedure (29) or from laboratory procedures and were excluded from the calculation on the prevalence of bacteraemia.

## Discussion

The results from this methodological investigation have shown that: (1) post-tooth brushing bacteraemia was only detected using culturing techniques (DAC and LC), although the isolates were not coincident both techniques were compared in the same subjects; (2) *F. nucleatum* was the only periodontal pathogen identified in blood, which was also detected as the most predominant bacteria in the subgingival samples from the same subjects; (3) bacteraemia induced by tooth brushing was mainly found in the gingivitis group, with a prevalence of 20% by DAC and 10% by LC; and (4) even though periodontitis subjects harbored significantly higher levels of periodontal bacteria in subgingival samples, these bacteria were not found in blood samples.

To the best of our knowledge, this is the first comparative study assessing different diagnostic methods (BACTEC, DAC, LC or qPCR) for detecting bacteraemia of oral bacteria in serum samples from the same subjects before and after tooth brushing. Only Kinane *et al.* (2005) reported bacteraemia induced by tooth brushing using more than one method in the same group (BACTEC and conventional PCR), indicating a greater sensitivity and specificity for PCR (11). The use of DAC has not been previously used in bacteraemia studies, although it is the gold standard in periodontal cultural microbiology for the analysis of the subgingival microbiota (21). In the present study, this method demonstrated the higher frequency of detection of bacteraemia.

In addition, data showed clear differences in the prevalence of bacteraemia depending on the diagnostic method used, which clearly highlight the importance of the selection of an appropriate technique for bacteraemia studies. No bacteria were detected by BACTEC and qPCR, 10% and 20% in the gingivitis group with LC and DAC, respectively, and 5.9% in the healthy group by DAC. This variability is, however, in agreement with previous studies that have reported prevalences of bacteraemia after tooth brushing ranging between 0 and 62% (8,10-14,17,18). The explanation of this variability was based on differences in the timing of the blood sample collection, the periodontal status of the subjects and the different microbiological diagnostic methods used in the different studies. In this investigation, the sampling conditions and the subject population were well characterized, what suggests that the variability may depend on the different microbiological diagnostic systems used to detect bacteraemia, what clearly limits the interpretation of this scientific evidence. Irrespective from the method used, the presence of periodontal pathogens in blood samples 30 seconds after tooth brushing was infrequent, what is in disagreement with similar studies collecting serum samples immediately after the triggering event (8,12,16).

The BACTEC system, which is even considered the gold standard to detect bacteraemia (30), was not able detect any bacteria in the present study. This fact could be explained by the difficulties in cultivating some fastidious periodontal bacteria, such as *P. gingivalis,* that may require more specific culture media or growth conditions, or that bacteria are rapidly degraded by the immune system and then culture is unable to detect them (19). In fact, no publication about tooth brushing induced-bacteraemia describes the presence of *P. gingivalis* by the BACTEC system. Similarly, qPCR is considered a very sensitive technique to detect bacterial DNA, but all blood samples analyzed in the present study were negative for *A. actinomycetemcomitans* and *P. gingivalis.* For *A. actinomycetemcomitans,* this was reasonable since these pathogens were also absent in the subgingival microbiota from the same patients. Also this bacterial species was not detected in most bacteraemia studies performed after tooth brushing (8,11-13,17). Although in one study the lack of detection of *A. actinomycetemcomitans* was attributed to the primers used (11), in the present study well validated specific primers were chosen (28). The lack of detection of *P. gingivalis* in blood by qPCR, in spite of being present in high numbers and proportions in the subgingival biofilm of mainly gingivitis and periodontitis subjects, is more difficult to explain, although other studies have also reported lack absence of *P. gingivalis* on bacteraemia samples (8,10-13,17).

The two microbiological approaches that were able to detect bacteraemia (DAC and LC), did not detect bacteria in the blood samples from the same patients. Interestingly, Kinane *et al.* (11) found similar inconsistent results, since they reported 13% (4/30) of positive bacteraemia using BACTEC and 23% (7/30) with conventional PCR. In the present study, both DAC and LC are culturing dependent techniques, but they use different enriched media to cultivate the blood sample: agar blood and Dentaid-1 media in DAC, and chocolate and Schaedler agar in LC. These differences in growth media, however, do not explain the discrepancy in the results since *F. nucleatum* was the only periodontal bacteria detected, in two patients by DAC and in one patient by LC. This presence of *F. nucleatum* in bacteraemia subjects is in accordance with its relative proportions in the subgingival samples, since it was the most prevalent bacteria in healthy and gingivitis group. *P. intermedia, F. nucleatum* and *P. gingivalis* were the most prevalent bacterial species in the chronic periodontitis group, which is consistent with the higher frequency of detection of *P. gingivalis* reported in Spanish patients with periodontitis (21). When compared with another post-tooth brushing bacteraemia study using BACTEC system (16), other periodontal bacteria, such as *A. actinomycetemcomitans,* were found in patients suffering periodontal disease. However, the bacterial species that have been isolated most frequently in post-tooth brushing blood cultures were *Streptococcus spp.* (45%), followed by obligate anaerobes (19%) and *Staphylococcus spp.* (15%). In the present study, *Staphylococcus spp., Bacillus spp., Corynebacterium spp., Micrococcus spp.,* and *Moraxella spp.* were also detected, but they were considered as likely contaminants, from the skin during blood extraction (29).

These results, however, should be interpreted with caution, due to some clear limitations, including the limited sample size, the absence of validation of the selected techniques and their detection limits, and the lack of a gold standard reference method. Despite these limitations, we can conclude that there is a disparity of the results depending on the microbial diagnostic technique used, what clearly suggests the need for proper validation of the methods to detect periodontal pathogens in bacteraemia before further studies are performed.

## References

[1] van Winkelhoff AJ, Rams TE, Slots J (1996). Systemic antibiotic therapy in periodontics. Periodontol 2000.

[2] Keijser BJ, Zaura E, Huse SM, van der Vossen JM, Schuren FH, Montijn RC (2008). Pyrosequencing analysis of the oral microflora of healthy adults. J Dent Res.

[3] Herzberg MC, Weyer MW (1998). Dental plaque, platelets, and cardiovascular diseases. Ann Periodontol.

[4] Tonetti MS, Van Dyke TE, workshop wgotj EA (2013). Periodontitis and atherosclerotic cardiovascular disease: consensus report of the Joint EFP/AAP Workshop on Periodontitis and Systemic Diseases. J Periodontol.

[5] Reyes L, Herrera D, Kozarov E, Roldá S, Progulske-Fox A (2013). Periodontal bacterial invasion and infection: contribution to atherosclerotic pathology. J Periodontol.

[6] Tomás I, Diz P, Tobías A, Scully C, Donos N (2012). Periodontal health status and bacteraemia from daily oral activities: systematic review/meta-analysis. J Clin Periodontol.

[7] Roberts GJ (1999). Dentists are innocent! "Everyday" bacteremia is the real culprit: a review and assessment of the evidence that dental surgical procedures are a principal cause of bacterial endocarditis in children. Pediatr Cardiol.

[8] Forner L, Larsen T, Kilian M, Holmstrup P (2006). Incidence of bacteremia after chewing, tooth brushing and scaling in individuals with periodontal inflammation. J Clin Periodontol.

[9] Fine DH, Furgang D, McKiernan M, Tereski-Bischio D, Ricci-Nittel D, Zhang P (2010). An investigation of the effect of an essential oil mouthrinse on induced bacteraemia: a pilot study. J Clin Periodontol.

[10] Lockhart PB, Brennan MT, Thornhill M, Michalowicz BS, Noll J, Bahrani-Mougeot FK (2009). Poor oral hygiene as a risk factor for infective endocarditis-related bacteremia. J Am Dent Assoc.

[11] Kinane DF, Riggio MP, Walker KF, MacKenzie D, Shearer B (2005). Bacteraemia following periodontal procedures. J Clin Periodontol.

[12] Sconyers JR, Crawford JJ, Moriarty JD (1973). Relationship of bacteremia to toothbrushing in patients with periodontitis. J Am Dent Assoc.

[13] Schlein RA, Kudlick EM, Reindorf CA, Gregory J, Royal GC (1991). Toothbrushing and transient bacteremia in patients undergoing orthodontic treatment. Am J Orthod Dentofacial Orthop.

[14] Bhanji S, Williams B, Sheller B, Elwood T, Mancl L (2002). Transient bacteremia induced by toothbrushing a comparison of the Sonicare toothbrush with a conventional toothbrush. Pediatr Dent.

[15] Crasta K, Daly CG, Mitchell D, Curtis B, Stewart D, Heitz-Mayfield LJ (2009). Bacteraemia due to dental flossing. J Clin Periodontol.

[16] Lockhart PB, Brennan MT, Sasser HC, Fox PC, Paster BJ, Bahrani-Mougeot FK (2008). Bacteremia associated with toothbrushing and dental extraction. Circulation.

[17] Hartzell JD, Torres D, Kim P, Wortmann G (2005). Incidence of bacteremia after routine tooth brushing. Am J Med Sci.

[18] Lucas VS, Gafan G, Dewhurst S, Roberts GJ (2008). Prevalence, intensity and nature of bacteraemia after toothbrushing. J Dent.

[19] Parahitiyawa NB, Jin LJ, Leung WK, Yam WC, Samaranayake LP (2009). Microbiology of odontogenic bacteremia: beyond endocarditis. Clin Microbiol Rev.

[20] Dorn GL, Smith K (1978). New centrifugation blood culture device. J Clin Microbiol.

[21] Sanz M, van Winkelhoff AJ, Herrera D, Dellemijn-Kippuw N, Simón R, Winkel E (2000). Differences in the composition of the subgingival microbiota of two periodontitis populations of different geographical origin. A comparison between Spain and The Netherlands. Eur J Oral Sci.

[22] Figuero E, Lindahl C, Marín MJ, Renvert S, Herrera D, Ohlsson O (2014). Quantification of periodontal pathogens in vascular, blood, and subgingival samples from patients with peripheral arterial disease or abdominal aortic aneurysms. J Periodontol.

[23] Armitage GC (1999). Development of a classification system for periodontal diseases and conditions. Ann Periodontol.

[24] Wikström M, Renvert S, Dahlén G, Johnsson T (1991). Variance in recovery of periodontitis-associated bacteria caused by sampling technique and laboratory processing. Oral Microbiol Immunol.

[25] Dahlén G, Renvert S, Wikström M, Egelberg J (1990). Reproducibility of microbiological samples from periodontal pockets. J Clin Periodontol.

[26] Alsina M, Olle E, Frias J (2001). Improved, low-cost selective culture medium for Actinobacillus actinomycetemcomitans. J Clin Microbiol.

[27] Boutaga K, van Winkelhoff AJ, Vandenbroucke-Grauls CM, Savelkoul PH (2003). Comparison of real-time PCR and culture for detection of Porphyromonas gingivalis in subgingival plaque samples. J Clin Microbiol.

[28] Boutaga K, van Winkelhoff AJ, Vandenbroucke-Grauls CM, Savelkoul PH (2005). Periodontal pathogens: a quantitative comparison of anaerobic culture and real-time PCR. FEMS Immunol Med Microbiol.

[29] McBryde ES, Tilse M, McCormack J (2005). Comparison of contamination rates of catheter-drawn and peripheral blood cultures. J Hosp Infect.

[30] Lafaurie GI, Mayorga-Fayad I, Torres MF, Castillo DM, Aya MR, Barón A (2007). Periodontopathic microorganisms in peripheric blood after scaling and root planing. J Clin Periodontol.

